# *Candida albicans* and *Candida dubliniensis* in Periodontitis in Adolescents and Young Adults

**DOI:** 10.1155/2022/4625368

**Published:** 2022-01-11

**Authors:** B. Jabri, M. Iken, S. Ait- Ou-amar, S. Rida, A. Bouziane, O. k. Ennibi

**Affiliations:** ^1^Research Laboratory in Oral Biology and Biotechnology, Faculty of Dental Medicine, Mohammed V University in Rabat, Rabat, Morocco; ^2^Clinical Biology Department, Faculty of Medicine and Pharmacy, Mohammed V University in Rabat, Rabat, Morocco

## Abstract

**Aim:**

This study aims to evaluate the association of *Candida albicans* and *Candida dubliniensis* with periodontitis in adolescents and young adults in a Moroccan population.

**Methods:**

426 subjects aged between 12 and 25 years were recruited for the study. A pool of plaque sample was taken. Samples were cultured on Sabouraud Chloramphenicol medium at 37°C for 24–48 hours and then identified by the Vitek 2 YST system. Clinical data and presence of *Candida albicans* and *Candida dubliniensis* were analyzed using Jamovi (Version 1.8).

**Results:**

*Candida albicans* was observed in 25 subjects among 68 diseased patients (37%) and in 60 subjects among 358 healthy patients (17%). It can be reported that under normal yeast conditions, there is a statistically significant difference between these two groups (*P* < 0.001). *Candida dubliniensis* was more prevalent in periodontitis than in healthy subjects (*P*=0.026). Regarding clinical variables, subgroups of periodontitis subjects showed significant statistical differences for periodontal probing depth, clinical attachment loss, and number of decayed teeth in advanced periodontitis in comparison with initial or mild periodontitis. The results also indicate that the presence of the two species of *Candida* is not related to gender or age (*P* > 0.05) nor related to the severity of the periodontal disease in this population.

**Conclusion:**

Within the limits of our study, *Candida albicans* is more frequently associated with periodontitis. The potential role of *C. albicans* in periodontitis pathogenesis is very complex. More studies on biofilm associated with different forms of periodontitis are necessary. It is also important to assess the coexistence of periodontitis and caries and the associated biofilms.

## 1. Introduction

Periodontal diseases are among the most common infectious diseases affecting the oral cavity. They are associated with the presence of microbial biofilm that affects the immune-inflammatory response. The unbalance between biofilm accumulation and host response causes the destruction of the periodontium, which can lead to the possible loss of teeth [1, 2]. The periodontal pocket is an elementary lesion of periodontitis and serves as reservoirs of microbial agents [3]. Microbiota associated with periodontitis is very complex and play a major role in the development and the severity of the disease. The associated microorganisms with periodontitis are both pathogenic and commensally species having a pathogenicity favored by environmental factors [4].

Several studies have reported that periodontal diseases are caused by highly virulent bacteria, that is, *Actinobacillus actinomycetemcomitans* (*Aa*) and *Porphyromonas gingivalis* (*Pg*) [5]. However, the role of other microorganisms such as yeasts is poorly known. Some data showed a prevalence of yeast up to 10 to 30% in periodontal pockets of healthy subjects [6, 7].

The most frequently isolated yeast from the oral cavity of healthy individuals is *Candida* with frequency of 31 to 55% [8]. Several species of this *Candida* genus have been identified in periodontal pockets from 7.1 to 19.6% of patients with periodontal disease [9, 10]. The most frequently associated with these diseases is *Candida albicans* yeast. Other less widespread species have been isolated too [11, 12], such as *Candida dubliniensis*, a yeast closely phylogenetically related to *Candida albicans*, with differences in genome sequencing [13].

This study aims to assess the association of *Candida albicans* and *Candida dubliniensis* with periodontitis among teenagers and young adults in a Moroccan population.

## 2. Materials and Methods

### 2.1. Study Population

The target population for this cross-sectional study was teenagers and young adults. The subjects were students from high schools and faculties randomly selected in the city of Rabat, Morocco.

Based on a frequency of *Candida* of 3.57% in healthy group and 26% in subjects with periodontitis (Uzura et al. (2008), a power of 95%, a risk *α* of 1%, and a ratio of cases to controls 1 : 5), the sample size estimation was 57 subjects in periodontitis group and 287 individuals in healthy group (a total of 344 subjects). We took into account the possible dropout cases in our study, so the sample size had been increased by 20%. Therefore, the global sample estimation was 413 subjects.

The research was done in accordance with the Ethical Principles for Medical Research involving Human Subjects outlined in the Declaration of Helsinki, and approval was obtained from the Biomedical Research Ethics Committee (CERB) of the Faculty of Medicine and Pharmacy of the Mohamed V University in Rabat under the number 102/19. We also asked for an authorization to access schools and faculties (authorization numbers: 18/8164; 19/0629; 19/6113).

All subjects were informed about the study and agreed to participate. The consents were signed by the students aging or above 18 years old and by the parents of those who were under 18.

Excluded students were those with infectious diseases, patients who had received periodontal treatment or had used an antibiotic during the last three months prior the plaque sampling, patients who had orthodontic treatment, and those who used tobacco or having diabetes.

### 2.2. Methods

#### 2.2.1. Periodontal Examination

Prior to the clinical examination, consenting students were interviewed using a structured questionnaire to collect demographic and behavioral data.

To assess the periodontal status, the following clinical variables were used: O'Leary plaque index (PI), bleeding on probing (BoP), full mouth periodontal probing (FMPP), and clinical attachment loss (CAL). Periodontal probing and attachment loss measurements were recorded at six sites per tooth, except third molars, using a North Carolina periodontal probe (Hu-Friedy, Chicago, IL, USA).

Based on the clinical examination, a subject was assigned a status of periodontitis or nonperiodontitis subject according to the 2017 World Workshop Classification of Periodontal Diseases and Conditions [14]. Periodontitis is defined as the presence of clinical attachment loss in at least 2 nonadjacent teeth or the presence of buccal or oral clinical attachment loss ≥3 mm with pocketing >3 mm in at least 2 teeth [15]. To address measurement errors, the threshold of CAL was set at 2 mm.

Moreover, stages and grades were also defined based on the severity and complexity of periodontal breakdown and based on the rate of periodontitis progression, respectively. For stages, the following criteria were used: Stage I, initial periodontitis where CAL at the site of greatest loss was 1 to 2 mm, there was no tooth loss due to periodontitis, and maximum probing depth was 4 mm; Stage II, moderate periodontitis in which CAL at the site of greatest loss was 3 to 4 mm, there was no tooth loss due to periodontitis, and maximum probing depth was 5 mm; Stage III, severe periodontitis with CAL at the site of greatest loss was ≥5 mm, probing depth was ≥6 mm, furcation involvement was Class II or III, tooth loss due to periodontitis was ≤4 teeth, and there was a risk of potential additional tooth loss; Stage IV, advanced periodontitis with CAL at the site of greatest loss was ≥5 mm, tooth loss due to periodontitis was ≥5 teeth, and there was risk of with extensive tooth loss and potential for loss of dentition.

For progression of periodontitis, grading was based on the estimation of importance of longitudinal data (CAL) as follows: Grade B, having low loss of attachment and periodontal destruction that commensurate with biofilm deposits; Grade C, CAL and periodontal destruction exceeds expectation given biofilm deposits; and specific clinical features evocative of periods of rapid progression and/or early onset disease (e.g., molar/incisor pattern).

Risk factors (smoking and diabetes) that may modify the grade were considered as exclusion criteria for this study.

The presence of *Candida albicans* was assessed in nonperiodontitis and periodontitis patients and in different subgroups of periodontitis subjects.

#### 2.2.2. Mycological Sampling

A pool of subgingival biofilm was randomized at six sites per patient, using an absorbent paper point inserted into the periodontal pocket or sulcus. The subgingival samples were transferred into an Eppendorf tube containing 2 ml of PBS (sodium chloride 137.0 mM NaCl, disodium phosphate 10.0 mM Na2HPO4, monopotassium phosphate 2.0 mM KH2PO4, and potassium chloride 2.7 mM KCl). The samples were then transported immediately to the laboratory for analysis. They were processed for yeast identification. Microbial culture and identification tests were performed at the Research Laboratory of Oral Biology and Biotechnology (LRBOB) Faculty of Dental medicine in Rabat, and the Parasitology-Mycology Laboratory of the Mohamed V Military Teaching Hospital, Rabat, Morocco.

#### 2.2.3. Identification of *Candida*

**Figure 1:**
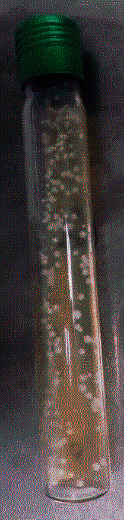
Macroscopic examination of the appearance of *Candida* on Sabouraud Chloramphenicol culture medium.

**Figure 2:**
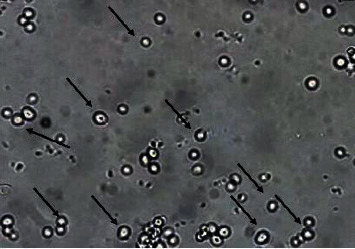
Appearance of Candida in a fresh state.

100 *μ*l of each pooled sample was aseptically inoculated into Petri dishes containing Sabouraud Chloramphenicol medium and incubated at 37°C for 24 to 48 hours [16]. The *Candida* colonies on this medium are creamy whitish and smooth (Figure 1) [17]. Under direct examination of the culture, the colonies appear as oval, ovoid, or elongated and possibly like budding elements (Figure 2) [18].

The colonies of *Candida* genus in each sample were numbered using the CFU/mL (colony forming units).

Identification by the Vitek 2 YST card system is as follows.

The Vitek 2 Yeast card was used according to the manufacturer's instructions by the Vitek 2 system (YST Insert Package: Ref 21343, Biomérieux, Inc.).

After a culture of 24 to 48 hours, the manipulation is minimized in a simple step of preparation, standardization, and dilution of the inoculum (Mc Farland: 1.80–2.20). The following strain, *Candida albicans* ATCC 90028, was used as a reference strain.

The cards, consisting of 64 wells with 47 biochemical tests, are automatically integrated, filled, sealed, and incubated at a temperature of 35°C.

The efficiency of the Vitek 2 Compact system is based on a colorimetric technique that is based on the reading of the cards every 15 minutes on the basis of three different wavelengths.

The time to obtain results of identification of yeasts is 18 hours.

#### 2.2.4. Data Analysis

Data analysis was conducted using Jamovi (Version 1.8) (The Jamovi Project, Sidney, Australia). Descriptive data were presented as a mean (±SD) and median and quartiles for quantitative measures and as a percentage for qualitative values.

Differences of clinical variables and *Candida albicans* and *Candida dubliniensis* status between nonperiodontitis patient and periodontitis subjects and between the subgroups of periodontitis groups were analyzed by parametric and nonparametric tests for variables expressed as median and quartiles.

The threshold of statistical significance retained was *P* < 0.05.

## 3. Results

A total of 426 students participated in the study. They were aged between 12 and 25 years old from both genders. The mean age of the population was 18,19 ± 3,20. The study population was divided mainly onto 2 groups: periodontitis subjects and nonperiodontitis subjects. Among 426 subjects, 16% had periodontitis. Demographic and clinical data of the population are summarized in Table 1.

The study population had globally very high plaque index and bleeding on probing index, even if the difference was statically significant between nonperiodontitis subjects and periodontitis subjects (Table 1).

*Candida dubliniensis* represented 4% of *Candida* genus in the cultivated samples, whereas *Candida albicans* represented 20%.

The comparison of the presence of *C. albicans* and *C. dubliniensis* regarding age and gender did not show significant statistical differences (*P* > 0.05) (Table 2).

When comparing periodontitis subgroups, accordingly to severity and rate of progression (stages and grades), there was no statistical differences for age (*P* > 0.05) (Tables 3 and 4).

Regarding clinical variables, subgroups of periodontitis subjects showed significant statistical differences for periodontal probing depth, clinical attachment loss, and number of decayed teeth in periodontitis Stage III and grade C in comparison with periodontitis Stage I or II and Grade B, respectively (Tables 3 and 4). The results also indicate that the presence of the two species of *Candida* is not related to the severity of the periodontal disease in this population (Tables 3 and 4).

## 4. Discussion

The current study found that 16% of the studied population aged between 12 and 25 years old had periodontitis. All over the world, studies suggested that the prevalence of periodontitis is 15–30% in adults, and sometimes higher [19–21]; among African adolescents, the prevalence of aggressive periodontitis was estimated as 3.4% to 6.5% [22, 23].

In a Moroccan population, Kissa et al. in 2016 found a prevalence of periodontitis of 11.3% (94/830) in students aged 12 to 25 [24]. However, in that study, the threshold of CAL was 5 mm, whereas the chosen threshold of CAL in our study was 2 mm to avoid underestimating the initial lesions of periodontitis. Data on the prevalence and determinants of periodontal disease, particularly in adolescents, helps the practitioner in diagnosing and managing cases before the onset of irreversible periodontal disease states.

Periodontal diseases are associated with several microorganisms of the subgingival plaque such as *Porphyromonas gingivalis* (*P.g.*) *Actinobacillus actinomycetemcomitans* (*A.a.*), *Prevotella intermedia,* and *Fusobacterium nucleatum* [25], but the role of oral yeasts in these diseases is not known until now. Compared to subjects with healthy periodontium, studies have reported an increased prevalence of *Candida* in periodontal pockets, particularly *Candida albicans*, in patients with periodontitis [6, 26]. However, these studies have been limited by microbial identification methods and/or small sample size.

Cuesta et al. [27] reported a value of 13% of the genus *Candida* of which 76.2% were *Candida albicans* in a population of 82 with 56 chronic periodontitis, which is confirmed by several studies and which also reported that *Candida albicans* was the most frequent species in periodontal pockets by 21.7% to 24.4% [12, 28].

According to previous studies, the distribution of *Candida albicans* isolates from the subgingival biofilm according to periodontal health status varied between 9,5% and 40% in subjects with chronic periodontitis, whereas in healthy subjects, the presence of *Candida albicans* was estimated between 3.8% and 36% [11, 26, 28–32] and 20% in patients with aggressive periodontitis [6]. In our study, *Candida albicans* was recovered in the periodontal pockets of 25 (37%) subjects with periodontitis versus 17% without periodontitis (Table 2).

The second species identified was *Candida dubliniensis*; this is a recently identified opportunistic pathogenic yeast associated with oral candidiasis, particularly in HIV-infected individuals. Few epidemiological studies have evaluated the prevalence of colonization by this species in periodontal pockets and its possible role in the oral environment of healthy and periodontitis patients [33]. A few studies have reported *Candida dubliniensis* colonization in the subgingival plaque of immunocompetent subjects with periodontal disease, with a prevalence of 4.4% [11] to 4.6% [28].

In this study, *Candida dubliniensis* colonizes periodontal pockets of 17 adolescents which represent 3.9% of the total population (Table 2).

Previous studies showed the presence of *Candida dubliniensis* between 2.5% and 7.7% in subjects with chronic periodontitis [6, 11, 28] 2.5% [26] and less frequently in healthy individuals (2.5%) [11, 12].

Jewtuchowicz et al. in 2009 [28] found that there was no statistically significant difference (*P* > 0.05) when studying *Candida dubliniensis* colonization in subgingival sites in immunocompetent individuals with periodontal disease and those with good periodontal health, while other authors did not find *Candida dubliniensis* in the gingival sulcus in healthy individuals [6, 26, 29, 34] nor in patients with chronic [29] and aggressive [6] periodontitis.

In our study, *Candida dubliniensis* was recovered from the periodontal pockets of 6 subjects with periodontitis (9%) versus 3% who did not have the disease (11 subjects) (Table 2).

We consider that these percentage differences could be related to geographical differences, as different etiologies of some fungal infections have been recognized according to geographical location and infectious niche [35, 36].

To analyze the degree of *Candida* colonization in the subgingival sites, the CFU variable was compared between the two groups. In general terms, the results showed a different distribution between the healthy and the sick; thus the sick group had a higher average CFU (197 [123–274] CFU/mL) compared to the control group (85 [46–123] CFU/mL) with a statistically significant difference between the two groups (*P* < 0.001) (Table 1); these results are corroborated by those of Ursula et al. in 2008 [6], while Janire et al. in 2018 found this difference between the groups but without statistical significance since the *P*=0.3 [32].

Researchers are interested in identifying this species in HIV-infected persons, especially in cases of oropharyngeal candidiasis, and for this reason, it can be concluded that it is an important human pathogen, although it is a rare constituent of the microbial flora of the normal human organism [34, 37], which is also confirmed by our study (rare species in patients with healthy and diseased periodontium) (Table 2).

Among the 426 subjects, women and men with *Candida albicans* germ were, respectively, 19% and 20% in this study and therefore the presence of this species is not related to sex (*P*=0.940) (Table 2), a result confirmed by several studies [10, 26, 38], although Reynaud in 2001 shows a slight difference in prevalence in women (20.3%) compared to men (8.2%) [10].

When analyzing periodontitis according to the severity and probable rate of progression, we noticed that there was no significant difference between stages or grades subgroups of periodontitis (Tables 3 and 4). However, we assume that the small sizes of subgroups may interfere with the results that we found. Canabaro et al. 2013 reported that *Candida albicans* was associated with severity of periodontitis [26].

In the present study, the median of decayed teeth was 2.0 [1.0–3.0], and when comparing this variable with the presence or absence of *Candida albicans*, we found a statistically significant difference (*P* < 0.05) (Table 2). This is corroborated with results which showed the high presence of *Candida albicans* in children with caries [39]. We also noticed that in periodontitis group, the number of decayed teeth was significant in comparison with periodontally healthy subjects.

This important observation was recently discussed by Nigrini et al. 2019, who reported that the physical interaction between *Candida* and some oral bacteria helps coadhesion within biofilms and promotes development chemical interactions that enhance the coexistence of this species [40].

The comparison of the average of decayed teeth of the students in our study between the two groups showed a statistically significant difference (*P* < 0.05), which can justify the relationship of the appearance of the two diseases.

The biofilm, where all the metabolic activities of these microorganisms take place, is one of the factors associated with the pathological progression and severity of oral diseases (caries and periodontitis), in addition to genetic factors, oral hygiene, individual and cultural behaviors and mode of nutrition, and factors related to host health, such as immunity and salivary secretion. In this regard, it can be concluded that the processes that lead to the appearance of caries and periodontitis are similar.

The microbial etiology of caries is related to polymicrobial infection of teeth and bacterial-fungal interactions. Several previous studies have explored the cariogenic role of *Candida* species and found that *Candida albicans* promotes biofilm formation and accumulation [41–43].

A synergistic interaction between *Candida albicans* and oral bacteria promoting the virulence of polymicrobial biofilms was reported [44, 45]. Indeed, regarding the specific case of periodontitis, the consumption of oxygen by *Candida albicans* seems to create an oxygen tension that helps *P. gingivalis* growth [46] and support *P. gingivalis* ability to invade host cells [47]. However, *A. actinomycetemcomitans*, bacteria strongly associated with periodontitis in teenagers and young adults, seems to inhibit fungal biofilm development [48].

Thus, the potential role of *Candida albicans* in periodontitis pathogenesis is very complex. More studies on biofilm associated with different forms of periodontitis are necessary. It is also important to assess the coexistence of periodontitis and caries and the associated biofilms.

## 5. Conclusion

In this study *Candida albicans* was more frequently isolated in periodontal pockets than *Candida dubliniensis.* However, further studies are required to understand the potential role of this yeast in the biofilm associated with periodontal disease. In this study, the number of decayed teeth was statistically significant in periodontitis patients. This observation pointed out the complexity of biofilms associated to periodontitis and caries, in which *Candida albicans* may have an influence on microbial colonization and coexistence.

## Figures and Tables

**Table 1:** Demographic and clinical characteristics and the presence of yeast in the nonperiodontitis and periodontitis groups.

	Nonperiodontitis subjects, *N* = 358 (84%)	Periodontitis subjects, *N* = 68 (16%)	*P* value
Gender (male/female)	198/160 55%/45%	36/32 53%/47%	0.719^(a)^
Age (years) (mean ± SD)	17.81 ± 3.20 [12–24]	20.22 ± 2.31 [14–25]	<0.001^(b)^
Plaque index (%)	100 [97–100]	100 [100–100]	0.001^(c)^
Bleeding on probing (BOP) (%)	86 [38–100]	100 [100–100 ]	<0.001^(c)^
Periodontal probing (FMPP) (mm)	2.17 [1.99–2.34]	2.52 [2.36–2.75]	<0.001^(c)^
Clinical attachment loss CAL (mm) ≥ 2 mm	-----	2.38 [2.00–3.42]	---------
Decayed teeth	2 [1–3]	4 [3–5]	<0.001^(c)^
Yeast CFU (/mL)	85 [46–123]	197 [123–274]	<0.001^(c)^

All clinical variables are expressed as median and quartiles, except gender and age. (a) Chi2; (b) Student's *t*; (c) Mann–Whitney.

**Table 2:** The presence of *Candida albicans* and *Candida dubliniensis* according to sex, age, and clinical variables.

		*Candida albicans*		*P* value	*Candida dubliniensis*		*P* value
		Absence *N* = 341	Presence *N* = 85		Absence *N* = 409	Presence *N* = 17	
Gender (*n*, %)	Female	154 (45.2%)	38 (44.7%)	0.940^(a)^	186 (45.5%)	6 (35.3%)	0.408^(a)^
	Male	187 (54.8%)	47 (55.3%)		223 (54.5%)	11 (64.7%)	
Age (years) (mean ± SD)		18.26 ± 3.17	17.95 ± 3.34	0.437^(b)^	18.16 ± 3.20	18.94 ± 3.19	0.328^(b)^
Periodontal probing (PP) (mm)		2.20 [2.00–2.40]	2.29 [2.09–2.53]	0.004^(c)^	2.22 [2.01–2.41]	2.28 [2.04–2.48]	0.364^(c)^
PPD >3 mm pocket depth		4.43 [4.25–5.00]	4.45 [4.20–4.73]	0.720^(c)^	4.40 [4.24–4.78]	4.50 [4.48–5.50]	0.990^(c)^
Clinical attachment loss CAL (mm) ≥ 2 mm		2.46 [2.00–3.60]	2.21 [2.00–3.00]	0.481^(c)^	2.38 [2.00–3.08]	2.78 [2.00–5.07]	0.415^(c)^
Decayed teeth		2 [1–3]	2 [1–4]	0.004 ^(c)^	2 [1–3]	2 [1–3]	0.701^(c)^
Presence of periodontitis		43 (63%)	25 (37%)	<0.001^(a)^	62 (91%)	6 (9%)	0.026^(a)^

All clinical variables are expressed as median and quartiles (nonparametric distributions), except gender and age. (a) Chi2; (b) Student's *t*; (c) Mann–Whitney.

**Table 3:** Comparison of age, clinical variables, and presence of *Candida albicans* and *Candida dubliniensis* according to stages in periodontitis group.

	Subgroup 1: Stage I, *N* = 27	Subgroup 2: Stage II, *N* = 33	Subgroup 3: Stage III, *N* = 8	*P* value
Age (years) (mean ± SD)	19.66 ± 2.33	20.33 ± 2.20	21.62 ± 2.32	0.101 ^(a)^
Periodontal probing (PP) (mm)	2.54 [2.32–2.72]	2.45 [2.36–2.72]	3.90 [2.58–5.73]	0.001 ^(b)^
Pocket depth> 3 mm	4.33 [4.20–4.50]	4.40 [4.25–4.76]	6.00 [4.78–8.37]	<0.001 ^(b)^
Clinical attachment loss CAL (mm) ≥ 2 mm	2.00 [2.00–2.00]	2.88 [2.44–3.50]	6.03 [4.45–7.75]	<0.001^(b)^
Presence of *Candida albicans*	11 (44%)	11 (44%)	3 (12%)	0.838 ^(c)^
Presence of *Candida dubliniensis*	3 (50%)	2 (33%)	1 (17%)	0.732^(c)^

All clinical variables are expressed as median and quartiles (nonparametric distributions), except gender and age. (a) ANOVA, (b) Kruskal–Wallis, and (c) Chi2.

**Table 4:** Comparison of age, clinical variables, and presence of *Candida albicans* and *Candida dubliniensis* according to grades in periodontitis group.

	Grade B, *N* = 45	Grade C, *N* = 23	*P* value
Age (years) (mean ± SD)	20.00 ± 2.13	20.65 ± 2.63	0.275 ^(a)^
Periodontal probing (PP) (mm)	2.48 [2.32–2.69]	2.66 [2.45–3.44]	0.002 ^(b)^
Pocket depth> 3 mm	4.33 [4.19–4.50]	5.00 [4.44–5.29]	<0.001 ^(b)^
Clinical attachment loss CAL (mm) ≥ 2 mm	2.00 [2.00–2.46]	3.86 [2.60–5.00]	<0.001 ^(b)^
Presence of *Candida albicans*	17 (68%)	08 (32%)	0.809 ^(c)^
Presence of *Candida dubliniensis*	4 (67%)	2 (33%)	0.979 ^(c)^

All clinical variables are expressed as median and quartiles (nonparametric distributions), except gender and age. (a) Student's *t*, (b) Mann–Whitney, and (c) Chi2.

## Data Availability

All data are included within the manuscript.
